# Supplementary material for: Interactome screening implicates BAG6 as a suppressor of UBQLN2 misfolding in ALS/FTD
Source: Front Mol Neurosci. 2026 Jan 5;18:1720347. doi: 10.3389/fnmol.2025.1720347 (PMC12813162; doi:10.3389/fnmol.2025.1720347)
Supplement: Supplementary file 1 [file Data_Sheet_1.docx]

# Interactome screening implicates BAG6 as a suppressor of UBQLN2 misfolding in ALS-dementia

Sang Hwa Kim*^1^, Claire E. Boos*^2^, Mark Scalf^2^, Akasha K. Wilkemeyer^1^ Lloyd M. Smith^2^ and Randal S. Tibbetts^1^

^1^Department of Human Oncology

University of Wisconsin School of Medicine and Public Health

1111 Highland Ave

Madison, WI 53705, USA

^2^Department of Chemistry

University of Wisconsin-Madison

1101 University Ave

Madison, WI 53706, USA

*These authors contributed equally this work

Corresponding author: Randal S. Tibbetts, Email: [rstibbetts@wisc.edu](mailto:rstibbetts@wisc.edu)

## Keywords: ALS, FTD, UBQLN2, BAG6, PEG10.

**Supplementary methods**

**GO Enrichment Analysis**

Differentially enriched proteins identified from the IP-MS datasets across all conditions (37°C iPSCs, 42°C iPSCs, and iMNs) were combined into a single input list for functional enrichment. Gene ontology enrichment analysis was performed using g:Profiler (https://biit.cs.ut.ee/gprofiler), with default settings and g:SCS multiple-testing correction. Enrichment results were exported both as a simplified visual summary and as a detailed table containing all significant GO terms and associated statistics. The complete output is provided as a CSV file.

**Quantitative PCR (qPCR)**

Total RNA was extracted from iPSCs using TRIzol reagent following standard procedures and reverse-transcribed into cDNA using the Bio-Rad cDNA synthesis kit according to the manufacturer’s instructions. Quantitative PCR was performed with gene-specific primers targeting PEG10, with GAPDH serving as the housekeeping control. Primer sequences were as follows:

PEG10 Forward: ACCACCAGGTAGATCCAACCGA

PEG10 Reverse: TGTCAGCGTAGTGACCTCCTGT

GAPDH Forward: GTCTCCTCTGACTTCAACAGCG

GAPDH Reverse: ACCACCCTGTTGCTGTAGCCAA

**Supplementary figure legends**

**Fig. S1. GO enrichment analysis of UBQLN2 interactome** **in iPSCs (37°C).** Proteins differentially enriched in UBQLN2 immunoprecipitations from iPSCs at 37°C were analyzed using g:Profiler. **(A)** Simplified visualization of significantly enriched GO biological processes, molecular functions, and cellular components derived from this combined dataset. **(B)** Full detailed enrichment output, including all GO terms, enrichment scores, corrected p-values, and term–protein associations.

**Fig. S2. GO enrichment analysis of UBQLN2 interactome in iPSCs under heat stress (42°C).** Proteins differentially enriched in iPSCs after heat stress (42°C, 1 h) were analyzed using g:Profiler. **(A)** Simplified visualization of significantly enriched GO biological processes, molecular functions, and cellular components derived from this combined dataset. **(B)** Full detailed enrichment output, including all GO terms, enrichment scores, corrected p-values, and term–protein associations.

**Fig. S3. GO enrichment analysis of UBQLN2 interactome in iMNs.** Proteins differentially enriched in UBQLN2 immunoprecipitations from iMNs were analyzed using g:Profiler. **(A)** Simplified visualization of significantly enriched GO biological processes, molecular functions, and cellular components derived from this combined dataset. **(B)** Full detailed enrichment output, including all GO terms, enrichment scores, corrected p-values, and term–protein associations.

**Fig. S4. PEG10 mRNA levels are not affected by UBQLN2 genotype or BAG6 knockdown.** PEG10 mRNA levels were measured by qPCR in iPSCs expressing UBQLN2^WT^, UBQLN2^P497H^, UBQLN2^4XALS^, or UBQLN2^I498X^, with or without BAG6 knockdown. Data are shown as mean ± SEM (n = 5).
