# Supplementary figures and images for: Interactome screening implicates BAG6 as a suppressor of UBQLN2 misfolding in ALS/FTD
Source: Front Mol Neurosci. 2026 Jan 5;18:1720347. doi: 10.3389/fnmol.2025.1720347 (PMC12813162; doi:10.3389/fnmol.2025.1720347)

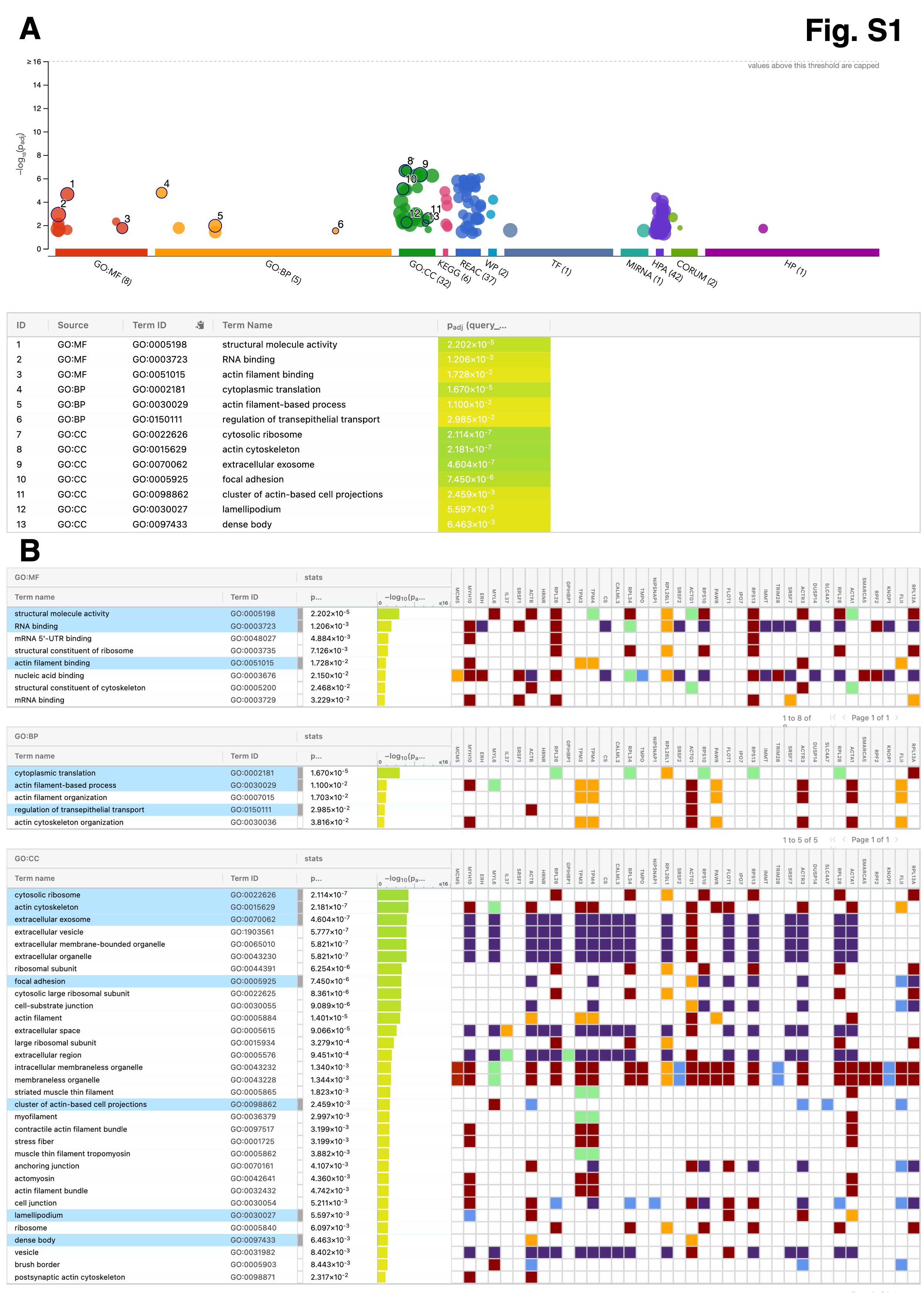

Supplement: Supplementary file 2 [file Image_1.jpg]

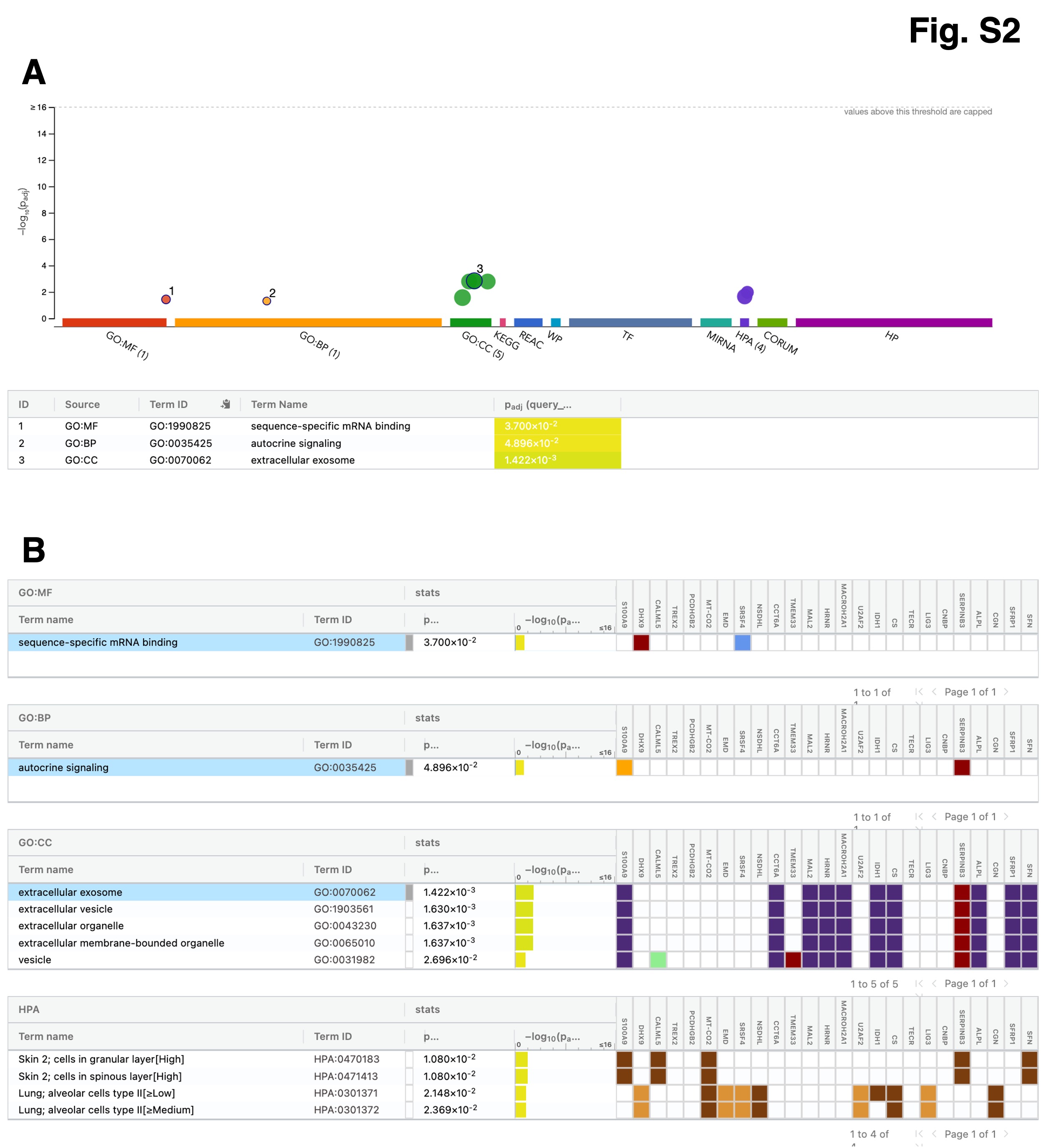

Supplement: Supplementary file 3 [file Image_2.jpg]

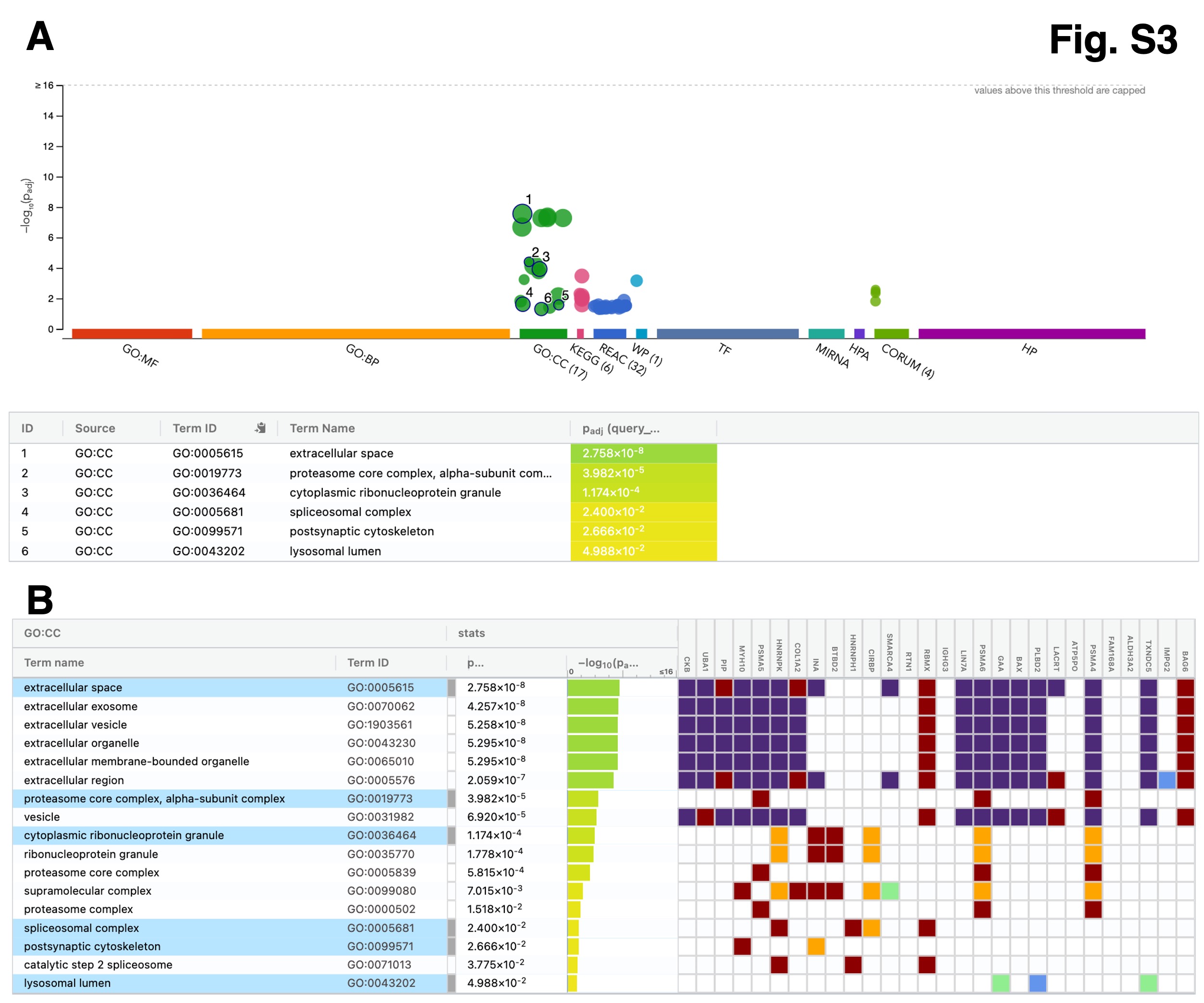

Supplement: Supplementary file 4 [file Image_3.jpg]

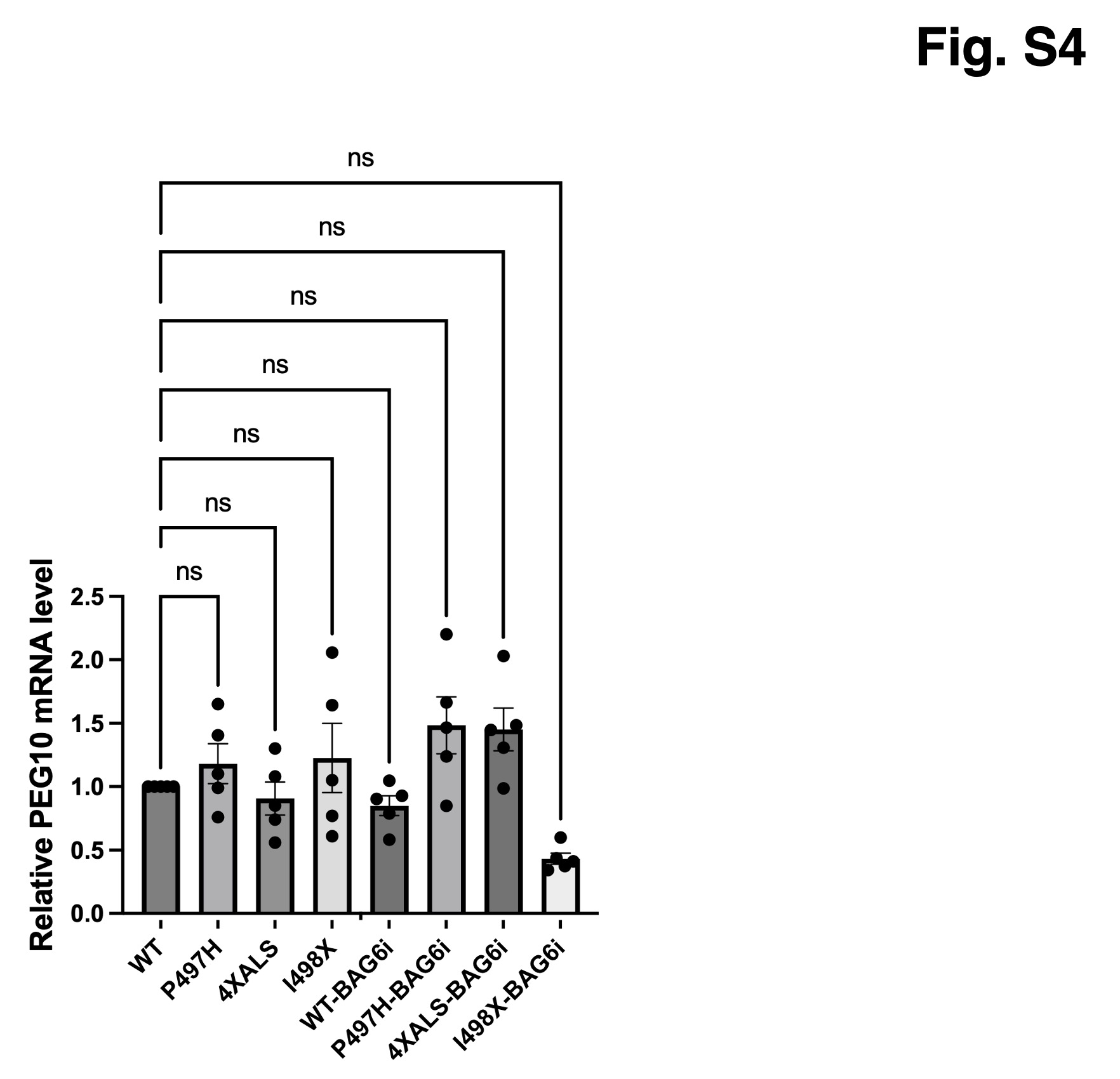

Supplement: Supplementary file 5 [file Image_4.jpg]
